# Targeting Mitochondrial Function with Chemoptogenetics

**DOI:** 10.3390/biomedicines10102459

**Published:** 2022-10-01

**Authors:** Amy Romesberg, Bennett Van Houten

**Affiliations:** 1 Department of Biological Sciences, College of Arts and Sciences, Carlow University, 3333 Fifth Avenue, Pittsburgh, PA 15213, USA; 2 UPMC Hillman Cancer Center, University of Pittsburgh, Pittsburgh, PA 15213, USA; 3 Department of Pharmacology and Chemical Biology, School of Medicine, University of Pittsburgh, Pittsburgh, PA 15213, USA

**Keywords:** mitochondrial DNA, chemoptogenetics, mitochondrial dysfunction, mitochondria, base excision repair, reactive oxygen species

## Abstract

Mitochondria are ATP-generating organelles in eukaryotic cells that produce reactive oxygen species (ROS) during oxidative phosphorylation (OXPHOS). Mitochondrial DNA (mtDNA) is packaged within nucleoids and, due to its close proximity to ROS production, endures oxidative base damage. This damage can be repaired by base excision repair (BER) within the mitochondria, or it can be degraded via exonucleases or mitophagy. Persistent mtDNA damage may drive the production of dysfunctional OXPHOS components that generate increased ROS, or OXPHOS components may be directly damaged by ROS, which then can cause more mtDNA damage and create a vicious cycle of ROS production and mitochondrial dysfunction. If mtDNA damage is left unrepaired, mtDNA mutations including deletions can result. The accumulation of mtDNA mutations has been associated with conditions ranging from the aging process to cancer and neurodegenerative conditions, but the sequence of events leading to mtDNA mutations and deletions is yet unknown. Researchers have utilized many systems and agents for generating ROS in mitochondria to observe the downstream effects on mtDNA, ROS, and mitochondrial function; yet, there are various drawbacks to these methodologies that limit their precision. Here, we describe a novel chemoptogenetic approach to target oxidative damage to mitochondria and mtDNA with a high spatial and temporal resolution so that the downstream effects of ROS-induced damage can be measured with a high precision in order to better understand the mechanism of mitochondrial dysfunction in aging, cancer, and neurodegenerative diseases.

## 1. Mitochondria, OXPHOS and ROS Production

Mitochondria are organelles in eukaryotic cells that generate the majority of ATP for the cell through a process called oxidative phosphorylation (OXPHOS). They also play important roles in programmed cell death, the biosynthesis of important metabolites, and the homeostasis of cellular iron and calcium. Mitochondria contain two membranes, and the inner of these two membranes has embedded within in it key protein complexes for OXPHOS. As glucose and other molecules, such as fatty acids, are catabolized by the cell, electrons from these molecules are carried by nicotinamide adenine dinucleotide (NAD) or flavine adenine dinucleotide (FAD) to the OXPHOS machinery on the inner mitochondrial membrane. NAD and FAD then deliver these electrons to complex I (NADH dehydrogenase) and complex II (succinate dehydrogenase), respectively, which are the first two complexes of the five complex electron transport chain (ETC). As electrons are passed to complexes I, III, and IV, protons are simultaneously pumped into the intermembrane space to form an electrochemical gradient. Complex V (ATP synthase) then utilizes this electrochemical gradient, spinning its nanoturbine to generate ATP from ADP + P_i_. Oxygen is the final electron accepter in the electron transport chain, and the addition of four electrons to the oxygen molecule creates metabolic water. The leakage of electrons from the ETC can generate superoxide radical anions, which can spontaneously or through the action of mitochondrial superoxide dismutase create hydrogen peroxide (H_2_O_2_). Superoxide can displace iron from iron sulfur centers in the ETC, inactivating key steps of OXPHOS. H_2_O_2_, if not broken down by glutathione peroxidase or peroxiredoxin, can interact with metal cations such as Fe^2+^ through Fenton chemistry, generating highly reactive carbonate and hydroxyl radicals [1]. These radicals can damage any macromolecule within mitochondria, including protein and lipid components and mitochondrial DNA (mtDNA).

## 2. MtDNA Packaging and Repair

Eukaryotic cells may contain up to a thousand mitochondria, and each mitochondrion possesses ten to fifteen copies of its own genome. The human mitochondrial genome is only ~16.5 kb in size, as opposed to the 3.3 billion base pairs of nuclear DNA, and encodes for 13 subunits of the OXPHOS machinery as well as two ribosomal RNAs and 22 transfer RNAs. The vast majority of the ~1500 enzymes and protein subunits that function in mitochondria are imported from the cytoplasm and mtDNA is replicated, transcribed, and translated within the organelle.

**Figure 1:**
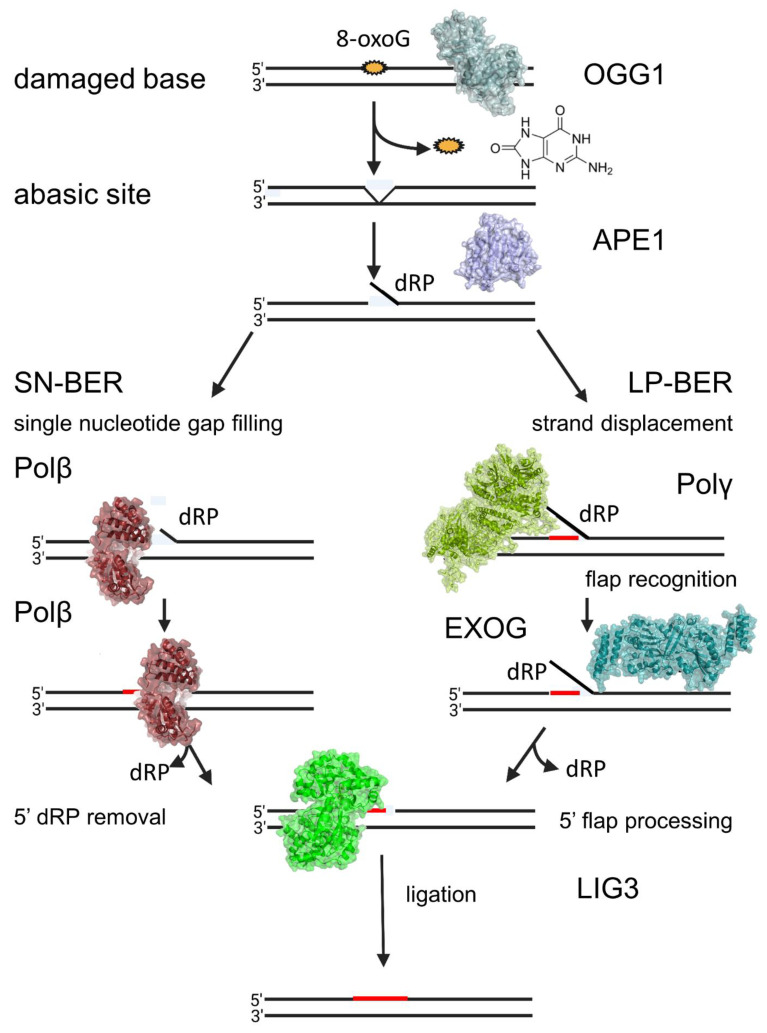
**Mitochondrial base excision repair pathway.** ROS damage in mtDNA leads to the production of the mutagenic 8-oxoguanine lesion, which is excised by 8-oxoguanine DNA glycosylase-1 (OGG1), PDB code: 1EBM, generating an abasic site. This abasic site is then processed by apurinic/apyrimidinic nuclease 1 (APE1), PDB code: 5DFF, which creates a strand break 5′ to the lesion. Repair of this strand break can proceed via either the single-nucleotide base excision repair (SN-BER) pathway (left) or the long patch base excision repair (LP-BER) pathway (right). In SN-BER, the nucleotide gap is filled by DNA Polymerase β (Polβ), PDB code: 1BPZ, and the 5′dRP is removed by Polβ’s dRP lyase activity. In LP-BER, DNA Polymerase γ (Polγ), PDB code: 5C53, displaces the flap and EXOG, PDB code: 5T40, recognizes and processes the flap. Polγ is also responsible for incorporating new nucleotides in LP-BER. The final step of base excision repair involves ligation of the sugar-phosphate backbone by DNA ligase III (LIG3), PDB code: 3L2P.

The mitochondrial genome is packaged into nucleoids [2] and due to its close proximity to the electron transport chain can endure increased spontaneous base damage as compared to the nuclear genome. These lesions, if not repaired, can cause transcriptional inhibition or replication errors and mutations [3]. The removal of oxidative and alkylation damage in the mtDNA is mediated by base excision repair (BER) (Figure 1). BER is the primary repair pathway for oxidized and alkylated mtDNA and involves the activity of several distinct enzymes that are encoded in the nucleus and imported into mitochondria [3]. In base excision repair, damaged bases are first recognized by a DNA glycosylase specific to that lesion. Some important DNA glycosylases that have been identified in mitochondria include 8-oxoguanine-DNA glycosylase (OGG1), which recognizes 8-oxoguanine, removes it, and cleaves the phosphodiester backbone at the lesion site, uracil DNA glycosylase (UNG1), which recognizes and removes uracil from DNA, and alkyladenine glycosylase (AAG), which recognizes primarily 3methylA, 7methylG, and ethenoA. After an abasic site has been generated by a glycosylase, the enzyme apurinic/apyrimidinic endonuclease 1 (APE1) cleaves the phosphodiester backbone [3]. Cleavage of the phosphodiester backbone results in a single-stranded break, which if allowed to persist, leads to apoptosis of mammalian cells [4]. The final two steps of base excision repair involve the incorporation of new nucleotides and the sealing of the phosphodiester backbone nick, which is performed by POLG and DNA ligase III [5], respectively. During long-patch BER in the mitochondria, POLG functions in strand displacement and EXOG processes the 5′ flap [4]. Most recently, DNA polymerase beta (Polβ) has been shown to function in mtDNA repair [6] (Figure 1).

Mouse models that seek to elucidate the mechanism of ROS-induced mitochondrial dysfunction have been created. Mice deficient in the BER glycosylase *Ogg1* were found to have accumulated 8-oxoG lesions in their mtDNA [7] and yet, their mtDNA showed no apparent increase in mutations. It is likely that the mtDNA molecules harboring these lesions are degraded, leading to a decrease in mtDNA copy number as a result [8]. Mice with a heterozygous knockout of the *Sod2* gene encoding for the mitochondrial antioxidant enzyme MnSOD display OXPHOS dysfunction, increased glycolysis and decreased ATP levels in their skin cells [9]. This indicates that increased ROS levels may be responsible for downstream mitochondrial dysfunction, but how? 

To elucidate the relationship of mtDNA damage and OXPHOS deficiency to disease states, several agents and systems have been employed in the past. Treating cells with H_2_O_2_ preferentially damages mtDNA over nDNA. H_2_O_2_ is uncharged and can diffuse freely within the cell, damaging components throughout the cell. Using an innovative QPCR assay to follow damage and repair in mtDNA we have shown that H_2_O_2_ induces three to 10-fold more damage to mtDNA than nDNA and protracted treatments suggested that the damage was unable to be repaired [10]. The use of glucose oxidase to generate a continuous stream of H_2_O_2_ in cells showed similar results, in irreparable mtDNA damage and a temporary decrease in mitochondrial function as evidenced by a decreased in the reduction in MTT [11]. A 2003 study by Santos et al. shows that after H_2_O_2_ treatment, the mitochondria exhibiting low mitochondrial membrane potential are those that also exhibit persistent mtDNA damage [12]. Treating mouse fibroblasts with H_2_O_2_ for 60 min leads to persistent mtDNA damage, loss of mtDNA copy number, and dysfunctional OXPHOS, implicating both damage and degradation of mtDNA in loss of mitochondrial function [13]. Studies using H_2_O_2_ suggest that mitochondria unable to repair oxidative mtDNA damage end up with OXPHOS dysfunction and organelle dysfunction in general, and yet similar levels of MMS-induced alkylation damage to mtDNA were able to be repaired efficiently. A major issue with using H_2_O_2_ to treat cells include the ability of the H_2_O_2_ to diffuse freely throughout the cell and potentially damage many targets, not just mtDNA [14]. 

Other agents have been applied to cells in order to generate mitochondrial ROS, including the complex I inhibitor rotenone and the neurotoxin MPTP (1-methyl-4-phenyl-1,2,3,6-tetrahydropyridine). When rotenone was applied to the growth medium of normal or DNA-repair deficient fibroblasts, increased intracellular ROS resulted, as evidenced by the oxidation of dihydroethidium to ethidium, but there was no detectable nDNA damage [15]. While the particular site(s) of rotenone-induced intracellular ROS cannot be determined by the dihydroethidium oxidation assay, which only serves as a quantitative and not a location-specific measurement of ROS [15], Brand and coworkers have mapped superoxide production to two sites in Complex I [16]. When the neurotoxin MPTP is applied to dopaminergic neurons, its metabolite MPP^+^ acts as a potent complex I inhibitor that results in the production of ROS and impairment of mitochondrial respiration [17]. In a separate study, MPTP was found to cause increased ROS production and extensive mtDNA damage in mouse brains, likely through oxidative stress from complex I inhibition [18]. Interestingly, cells that inactivate complex I completely are better able to maintain their mitochondrial membrane potential and keep ROS levels low, indicating that complex I dysfunction is a major source of mitochondrial ROS [19].

Base damage can also cause replication blocking lesions, which may be overcome by DNA polymerase ϴ translesion synthesis, which might be a source of mtDNA mutations [20]. Replication errors are also associated with mtDNA deletions [3].

## 3. Overview

MtDNA damage and dysfunction have been implicated in aging, cancer, and neurodegenerative diseases such as Parkinson’s disease and Alzheimer’s disease. Mitochondrial dysfunction in many of these conditions may take decades to appear, which is difficult to reproduce in a lab. Rather, researchers have used many tools and damaging agents in an attempt to replicate the initial insult responsible for the pathogenesis of these conditions, and yet each of these methods has benefits and drawbacks. Data suggest that persistent oxidative damage to mtDNA leads to a downstream dysfunction in OXPHOS leading to a vicious cycle of ROS production and mtDNA damage. This review discusses the various historical methods of replicating mitochondrial dysfunction in the laboratory and introduces a newly developed chemoptogenetic approach to specifically target mtDNA and thus better understand the role of oxidative mtDNA damage in these conditions.

## 4. Chemoptogenetics and Mitochondrial Injury

Mitochondria-targeting drug conjugates have also been developed to selectively damage this organelle. These include mitochondria-penetrating peptides (MPPs), which accumulate inside mammalian mitochondria and can be conjugated with DNA-damaging agents such as chlorambucil and doxorubicin [14,21,22,23]. Wisnovsky and colleagues utilized the chemical probe mt-Ox, a DNA oxidizing agent that is targeted to mitochondria, to selectively damage mtDNA [14]. Mt-Ox contains a highly fluorescent chromophore, thiazole orange, that generates singlet oxygen in response to visible light and functions at maximum efficiency in green light (500 nm), which limits its penetration into deep tissues, in addition to requiring a strict adherence to a dark environment for animals or cells immediately following treatment [24]. In addition, the mitochondrial entry of the cationic mt-Ox may be influenced by alterations in mitochondrial membrane potential and the hydrophobicity of the mitochondrial inner membrane, and in fact, it has been argued that lipophilic cations may not reach the mitochondrial matrix at all [24,25,26]. 

Photosensitizing agents have been used to induce targeted damage to mitochondria and has been the basis of photodynamic therapy [27]. When photosensitizer molecules are exposed to specific wavelengths of light, they produce ROS, and these photosensitizers are expressed or introduced to living cells so that researchers can deliver ROS to specific targets within cells [28]. Previous photosensitizers include KillerRed, a red fluorescent protein, that when irradiated with green light, is able to generate ROS at levels sufficient to kill both prokaryotic and eukaryotic cells. This protein can be expressed in cells under various promotors or can be fused to a localization signal or protein to generate large amounts of ROS at a particular time and place at levels far higher than other photosensitizing agents such as DsRed [29]. KillerRed has been expressed in zebrafish to target membranes in neurons and cardiac muscle and has been shown to induce the translocation of mitophagy proteins such as Parkin [30,31,32]. One major limitation of KillerRed includes the reported toxicity of the KillerRed transgene, even when the light has not been applied [28,29]. Another genetically encoded photosensitizing agent, miniSOG (mini Singlet Oxygen Generator), has been used for cell ablation by targeting the outer mitochondrial membrane of neurons in *C. elegans* [33]. MiniSOG uses blue light exposure for long periods (0.5–1.5 h) to generate ROS, and this long light exposure is a major limitation of the technology because it does not allow for the precise temporal production of ROS [33]. In addition, blue light itself is toxic to species such as *C. elegans* and may result in cell death in the absence of miniSOG activity [33]. That mitochondria can be spatially and temporally targeted by a photosensitizing agent has driven the field forward in elucidating the specific events after initial ROS-induced damage to cellular components, and yet there are some drawbacks to previously developed photosensitizers. One major disadvantage of KillerRed and mini-SOG is in their requirement for high intensity light for inactivation, in addition to their ability to generate ROS in the absence of the photosensitizer protein due to biological chromophores producing light that overlaps their spectra [28].

**Figure 2:**
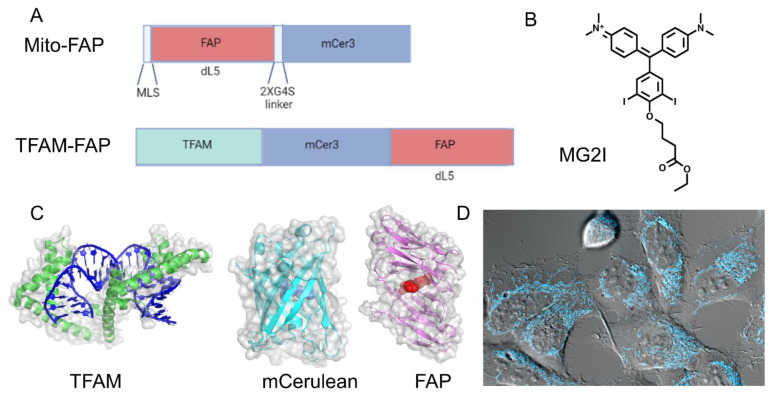
**Mitochondrial-targeting chemoptogenetics constructs.** We have created a highly controlled system that can help to elucidate the downstream effects of ROS damage within mitochondria. (**A**) The structure of the Mito-FAP and TFAM-FAP constructs. (**B**) The structure of the MG2I dye, that when bound by FAP and exposed to near-infrared wavelengths (642–660 nm), generates singlet oxygen. (**C**) The structure of several molecules in our chemoptogenetics system. TFAM is closely associated with mtDNA and upon fusion to the mCerulean-FAP construct, targets the ensuing singlet oxygen to mtDNA. mCerulean is a constitutively active fluorescent protein that allows one to visualize the subcellular location of the construct. The fluorogen-activating protein (FAP) is bound to the MG2I dye, which in the presence of near-infrared light, generates ROS as described above. (**D**) HEK293 cells stably transfected with TFAM-FAP, visualized within mitochondria via mCerulean fluorescence.

To overcome these problems and to understand the spatiotemporal events leading to mitochondrial dysfunction, we designed a precise, highly controlled chemoptogenetic tool to generate mitochondrial damage that is based on the FAP (Fluorogen Activating Protein) approach, as developed by Professor Marcel Bruchez [28] (Figure 2). The basis of this technology is a halogenated malachite green dye (MG2I) which is non-fluorescent until it is bound by the FAP. Once bound, the MG2I becomes highly fluorescent to near-infrared wavelengths (642–660 nm), and causes the production of singlet oxygen in the excited state. Singlet oxygen is highly reactive with a half-life of four μs, and can damage all macromolecules within a 200 nm radius. This chemoptogenetic tool allows for precise spatial and temporal control of ROS production, high efficiency for generating ROS, increased photostability, and absorption at near-infrared wavelengths (642–660 nm) for increased tissue penetration and simultaneous in vivo visualization. In addition to requiring less light dosage for more precise targeting, the free dye in the FAP system is unable to produce measurable fluorescence or singlet oxygen, which distinguishes it from other optogenetic and dye-targeting approaches. 

**Figure 3:**
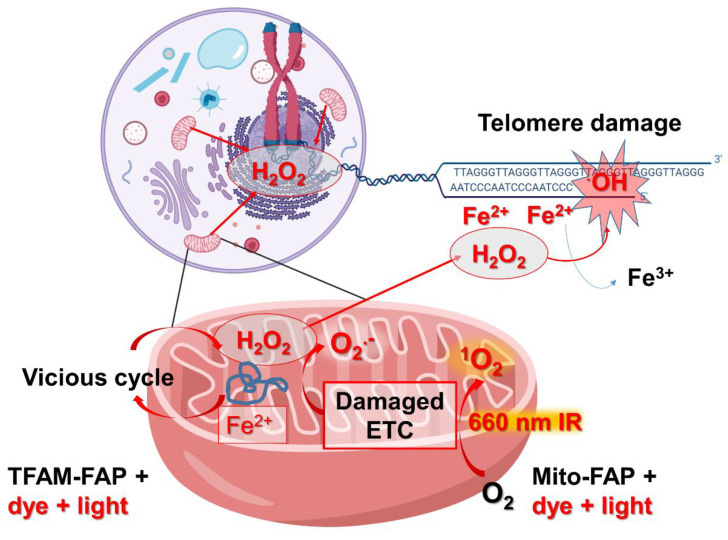
**Dysfunctional mitochondria cause a vicious cycle of ROS production that preferentially damages mtDNA and telomeres.** Upon binding to the MG2I dye and exposure to near-infrared light at 660 nm, Mito-FAP and TFAM-FAP generate singlet oxygen (^1^O_2_) with high quantum yield in close proximity to the electron transport chain (ETC) and the mtDNA, respectively. Oxidative damage to ETC components leads to a secondary wave of superoxide (O_2_^●−^), which is converted to H_2_O_2_ by manganese superoxide dismutase (MnSOD). Likewise, persistent oxidative damage to mtDNA can lead to ETC dysfunction and eventual H_2_O_2_ production within mitochondria. This H_2_O_2_ can freely diffuse throughout the cell and can be converted to the highly reactive hydroxyl radical (^●^OH) via interaction with iron (Fe^2+^) associated with the mitochondrial matrix or telomeric sequences, respectively. The hydroxyl radical can then damage mtDNA and telomeric sequences to lead to a vicious cycle of mitochondrial ROS production and premature aging, respectively.

Our group has used the mito-FAP system to target singlet oxygen damage to mitochondria [34]. The construct we used consisted of FAP fused to the mitochondrial leading sequence of COXIV and COXVIII that targeted the protein fusion to the mitochondrial matrix (Figure 2). We utilized HEK293 cells that stably expressed Mito-FAP in a recent study addressing whether dysfunctional mitochondria produce ROS that target other subcellular components. When combined with 50 nM MG2I dye activated with five minutes of NIR light at 660 nm, singlet oxygen was generated in the mitochondrial matrix. We then looked at several endpoints at four h and 24 h, and immediately following the dye and light treatment, including examining several mitochondrial and extramitochondrial targets. The mitochondrial endpoints measured in our study included mitochondrial OXPHOS, ETC complex activity and expression, mitochondrial membrane potential, mitochondrial dynamics, mtDNA damage, and mitochondrial ROS production [34] (Figure 3). 

At four hours post-treatment, there was a reduction in oxygen consumption and a concomitant increase in glycolysis indicating mitochondrial OXPHOS dysfunction. No change in mitochondrial bioenergetics was seen in cells that did not express Mito-FAP, indicating that the Mito-FAP construct, in combination with MG2I dye and light, was responsible for the change in bioenergetics. The presence of the singlet oxygen quencher sodium azide during light activation or the ROS scavenger N-acetylcysteine (NAC) during and after light activation was able to partially or completely protect mitochondrial respiration, respectively, indicating that ROS production was causative for mitochondrial dysfunction. Additionally, there was a progressive decline in the activity of several mitochondrial ETC complexes, including complexes I, III, and IV beginning at four hours post-treatment. Western blot indicated a decrease in Complex IV subunit II and Complex I subunit NDUFS3, both nuclear-encoded ETC genes [34] (Figure 3).

To determine whether ROS generation in the mitochondria leads to a change in mitochondrial membrane potential, the cationic fluorescent probe tetraethylbenzimidazolylcarbocyanine iodide (JC-1) was used 24 h after the dye and light treatment. A red-to-green shift of JC-1 fluorescence is indicative of a loss of mitochondrial membrane potential, and nearly a third of the Mito-FAP cells with dye and light treatment displayed this shift. In addition to a loss of mitochondrial membrane potential, fragmented mitochondria were observed four hours after treatment, and levels of the phosphorylated mitochondrial fission protein Drp1 increased at 24 h following treatment, indicating increased activity of this protein. Mitochondrial fission has been proposed as a way for the cell to separate any remaining functional mitochondria from irreversibly damaged ones that are destined for mitophagy. In addition to the effects on OXPHOS, mitochondrial dynamics and membrane potential, and ETC complex activity and expression, the dye and light treatment resulted in mtDNA damage that persisted for at least 24 h after the treatment, in contrast to a lack of nDNA damage [34].

Protection of loss of mitochondrial function after light and dye treatment of Mito-FAP expressing cells by N-acetylcysteine suggested that ROS-damaged mitochondria generate another wave of ROS that amplifies damage to the mitochondria and to the cell. MitoSOX dye indicated superoxide production in nearly 80% of cells at four hours post-treatment, and this increased superoxide signal persisted for at least 48 h post-treatment. As superoxide is quickly converted to the freely diffusible H_2_O_2_ by MnSOD within the mitochondria, we next explored the nature and severity of any extramitochondrial damage resulting from this secondary wave of ROS, including effects on cell growth, nuclear proteins, and telomeres.

Cells treated with dye and light showed a significant reduction in cell growth that was not due to apoptosis but instead was caused by cell cycle delay driven by DNA replication stress. If dysfunctional mitochondria generate significant fluxes of H_2_O_2_, then it might be expected that nuclear proteins and DNA could be damaged. To determine the downstream effects of mitochondrial ROS on the nucleus, we used a ratiometic approach to detect oxidized versus free thiols and determined that nuclear proteins were oxidized by H_2_O_2_, in combination with an increase in the pHyper-nuc signal, a fluorescent nuclear H_2_O_2_ sensor. Surprisingly, we could not detect gross nuclear DNA damage using the alkali comet assay. However, we found that the dysfunctional mitochondrially generated H_2_O_2_ causes a large increase in telomere damage. This may be due to the fact that telomeric sequences readily bind iron and thus are much more susceptible to Fenton chemistry and subsequent hydroxyl radical formation [35]. Forty-eight hours post-treatment, we measured the colocalization of a telomere probe to 53BP1, a double-strand break repair protein, to identify double-strand breaks in telomeres. We found a two-fold increase in these foci with no increase in double-strand breaks elsewhere in nDNA, leading to the conclusion that mitochondrially generated H_2_O_2_ preferentially damages telomeres [34] (Figure 3). These data support the mitochondrial theory of aging, in that dysfunctional mitochondrial generate ROS that damage telomeres, possibly leading to premature cellular senescence and death [36].

Chemoptogenetic studies involving the Mito-FAP fusion have been important for elucidating the downstream effects of delivering a single dose of singlet oxygen to mitochondria, and yet, the identity of the primary ROS target(s) in mitochondria that leads to vicious cycles of ROS production and mitochondrial dysfunction in aging and disease processes remains unsolved. One aspect of the Mito-FAP fusion construct is that while a cytochrome oxidase leader sequence was used, the precise mitochondrial matrix location was not known.

The mito-FAP system has been extended to ablate mitochondrial function in living zebrafish neurons by Dr. Ed Burton’s laboratory [37]. Double transgenic (NeuMito-FAP) zebrafish were generated to only express Mito-FAP in their neuronal mitochondria. When these zebrafish larvae were treated with dye and light, there was an acute loss of motor function in these larvae but not in controls: these affected zebrafish lost their ability to swim. This loss of visual-motor function did not recover, even after four days. Whole-cell patch-clamp recordings were then performed during light exposure to understand the mechanism of neurological dysfunction, and it was found that sensory neurons in the NeuMito-FAP-MG2I larvae were depolarized during the dye plus near-infrared light exposure, from −60.8 mV to −29.4 mV, leading to an increased refractory period. To determine if a lack of ATP in neurons caused the depolarization, phosphocreatine, which through the action of phosphocreatine kinase can resupply ATP, was added to the patch pipette solution, and the depolarization was circumvented. This indicates that the dye plus light treatment in the mitochondria of these NeuMito-FAP zebrafish larvae led to decreased ATP and a decline in the activity of ATP-dependent pumps such as the Na^+^/K^+^ ATPase and neuronal depolarization. A Seahorse flux analyzer was used to measure mitochondrial respiration in paralyzed whole larvae exposed to near-infrared light, and a 24% decrease in oxygen consumption rate was observed in the NeuMito-FAP-MG2I larvae compared to controls. Next, the brain cells of the NeuMito-FAP-MG2I larvae were evaluated in the flux analyzer during and after light treatment, and they showed a dramatic decrease in maximal respiration compared to controls. The loss of basal oxygen consumption and maximal respiration shows that the neuronal mitochondria of the NeuMito-FAP-MG2I were disrupted by the treatment [37]. This study demonstrated a clear link between mitochondrial dysfunction and progressive neuronal depolarization leading to increased neuronal cell death, which is a potential mechanism of action for the pathogenesis of specific neuronal cell populations in neurodegenerative diseases such as Parkinson’s disease and Alzheimer’s disease.

## 5. Chemoptogenetics and MtDNA

**Figure 4:**
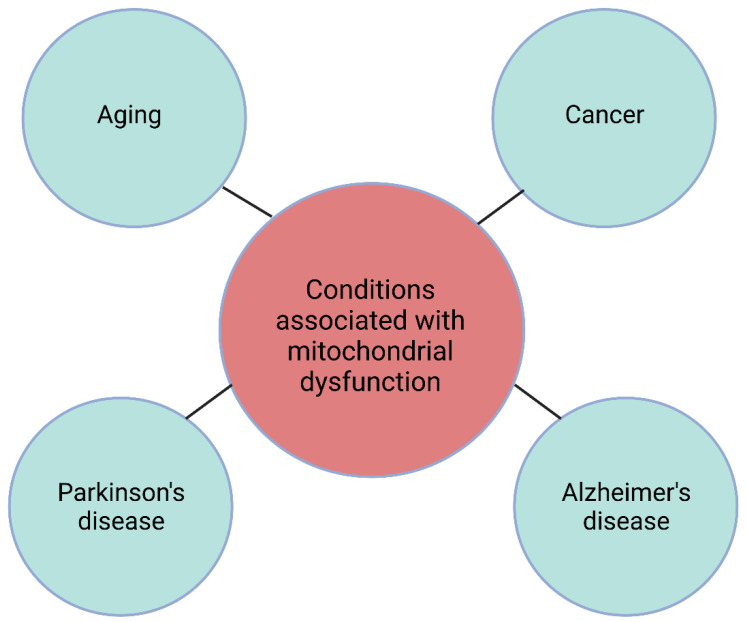
**Conditions associated with mitochondrial dysfunction.** An accumulation of deletions of mtDNA is observed in Parkinson’s disease patients, Alzheimer’s disease patients, and in the normal aging process, and abnormal mitochondrial structure and function have been observed in the brains of Alzheimer’s patients. Parkinson’s and Alzheimer’s tissues also show a decrease in absolute mtDNA copy number, indicating that damaged mitochondrial genome copies are not being sufficiently restored to normal levels in these conditions. Mitochondrial mutations have been linked to a wide variety of cancers and appear to drive metastasis through accelerated mutagenesis driven by mitochondrial dysfunction and ROS production.

In order to better understand the precise role that mtDNA damage plays in mitochondrial dysfunction in cancer, neurodegeneration, and aging, it is imperative that new methods are developed to specifically target mtDNA and not nuclear DNA or other macromolecule components (Figure 4). Mitochondrial transcription factor A (TFAM) is the most abundant structural component of mitochondrial nucleoids [38,39]. Due to its tight association with mtDNA, TFAM provides an opportunity to create specific mtDNA damage from singlet oxygen using the FAP-dye light approach. We have therefore fused FAP to the c-terminus of TFAM. Singlet oxygen produces almost exclusively 8-oxoguanine (8-oxoG) damage in DNA, and using this approach we can for the first time introduce 8-oxoG directly into mtDNA (Figure 3).

Preliminary studies in our lab show that a single treatment with dye and 660 nm light reduces the growth of our HEK293 TFAM-FAP cells, as measured by CyQuant. We have also observed mitochondrial dysfunction after multiple treatments with light plus dye, specifically, uncoupled mitochondrial function as measured in the Seahorse extracellular flux analyzer. Future studies involve quantifying the level of 8-oxoG lesions in mtDNA with quantitative long-amplicon PCR [14] following dye + light treatment. With the precise temporal and spatial control of the TFAM-FAP system, we can subject mtDNA to different levels and patterns of ROS exposure to answer the questions asked previously in this article: namely, are dysfunctional and/or damaged OXPHOS components generating ROS that then preferentially target mtDNA, or is damaged mtDNA leading to damaged OXPHOS components that then generate a vicious cycle of increased ROS and mtDNA damage? What is the nature and severity of mtDNA damage that leads mitochondria to degrade their DNA rather than repair it, and what does this mean for the pathogenesis of neurodegenerative diseases and cancer? These questions are placed in the context of what is known with regard to several human pathologies associated with mitochondrial dysfunction in the Section 6.

## 6. MtDNA Mutations and Pathologies

An accumulation of mtDNA deletions has been observed in older people, Parkinson’s disease (PD) patients, and Alzheimer’s disease (AD) patients [40]. The substantia nigra of PD patients and older individuals exhibited clonal expansion of deleted mtDNA, and neurons with cytochrome c oxidase (COX) deficiency showed significantly more mtDNA deletions than COX-proficient neurons [40] (Figure 4). A search for common mtDNA point mutations in the affected brain tissue of Parkinson’s and Alzheimer’s patients revealed that the A4336G mutation in a mitochondrial tRNA gene was present at low levels in Alzheimer’s and Parkinson’s patients but not in age-matched controls [41], suggesting that mtDNA deletions are more predictive of neurodegenerative conditions than mtDNA mutations. With regard to other neurodegenerative conditions, several studies have observed abnormal mitochondria [42], COX deficiency, and OXPHOS defects [43] in the brains of AD patients. A 2004 study by Coskun et al. suggested that mtDNA control region mutations as well as loss of mtDNA copy number could explain AD-related mitochondrial deficiency [44].

Mutations in mtDNA have also been linked to carcinogenesis and are found in a wide variety of cancers [45,46]. Interestingly, the metastatic nature of these cells could be prevented by pretreating the cells with a ROS scavenger, suggesting that cancers may select for mutations that drive further mtDNA mutation and dysfunction [47,48] (Figure 4). Later studies have reported similar results, with mutations in genes that result in complex I deficiency in particular appearing to drive metastasis [49,50,51,52]. A 2016 study by Gitschlag et al. hypothesized that mutations causing OXPHOS deficiency and increased ROS production induce an unfolded protein response (UPR) in mitochondria (UPR^mt^). The UPR^mt^ has several downstream mediators, including mitochondrial sirtuin SIRT3, which activates FOXO3a, followed by the activation of antioxidants including manganese superoxide dismutase (SOD2) and the removal of badly damaged mitochondria via mitophagy [53]. The induction of the UPR^mt^ has been suggested to lead to mitochondrial biogenesis and replication that is not selective for particular mtDNA molecules, allowing for wild-type and mutant mtDNA genomes to be replicated and for the vicious cycle to continue [54]. Even so, the particular mtDNA mutation(s) that are able to activate the UPR^mt^ are yet unknown [55], and so it is imperative that researchers have a mechanism by which damage can be conferred to mtDNA in particular to identify particular genes or mutations that drive carcinogenesis and metastasis.

The concept that mtDNA mutations are driven primarily by oxidative damage has been challenged more recently. A 2020 study by Yuan et al. performed the whole-genome sequencing of mtDNA from malignant tumors and from aging tissues and proposed that it is, in fact, errors in mtDNA replication, and not oxidative lesions, that are the primary cause of mtDNA mutations in malignant tumors. This was namely due to their finding mutations that were predominantly strand-asymmetric substitutions of C to T and of T to C, rather than the major ROS-induced mutation of G to T in these tumor tissues, as driven by the 8-oxoG lesion [8]. However, patient survivorship bias in carcinogenesis should be considered for the selection of specific genotypes. This same study found enormous variability in mtDNA copy number between cancer types and even within the same tissue type from different patient samples, and yet these copy number variables were not directly compared with the different types of mtDNA mutations, but rather by cancer type, patient age, truncating vs. non-truncating mutations, and cancer stage [8]. Our earlier studies with oxidized mtDNA show a precipitous drop in mtDNA copy number as a result of oxidative stress, and so it is entirely possible that the mutations seen in these tumors are merely the surviving mutations after many cell divisions and are not necessarily the drivers of carcinogenesis [13]. Recent studies propose that absolute mtDNA copy number is as important as the proportion of mutated mtDNA copies in determining the pathogenesis of cancer and neurodegenerative diseases [56]. An increase in absolute mtDNA copy number has been proposed as a mechanism by which cells with dysfunctional mitochondria sustain respiratory activity, and when this compensatory mechanism does not function appropriately, a disease state results [56]. For example, the dopaminergic neurons of Parkinson’s disease patients do not show a compensatory increase in absolute mtDNA copy number in response to increased mtDNA deletions [57], and show a reduction in mtDNA content in general [58]. Alzheimer’s patient frontal lobe tissue shows a significant reduction in mtDNA content when compared with controls [44,59]. The link between mtDNA copy number and cancer is less clear, in that the risk of many cancers correlate with increased mtDNA copy number, while the risk for other cancers correlate with lower mtDNA copy number [56].

Studies that detect mtDNA deletions and/or mutations in diseased tissues of patients are unable to elucidate the mechanism of the damage leading to these disease states, but rather only provide information about the outcome. Thus, these retrospective studies, while informative, lack clear cause and effect relationships, and three key questions need to be explored: (1) do dysfunctional and/or damaged OXPHOS components generate ROS that then preferentially target mtDNA, (2) does damaged mtDNA lead to damaged OXPHOS components that then generate a vicious cycle of increased ROS and mtDNA damage, and (3) what is the nature and severity of mtDNA damage that leads mitochondria to degrade their DNA rather than repair it?

To directly address these questions, we require a system that can specifically and precisely oxidize mtDNA and not nuclear DNA. It is also imperative to understand what particular aspects of mtDNA damage induce the degradation versus the repair of mtDNA lesions in order to elucidate how this decision impacts the pathogenesis of neurodegenerative diseases and cancers.

The singlet oxygen generated by the chemoptogenetic system produces almost exclusively 8-oxoG damage in DNA. By coupling the chemoptogenetic system to TFAM, an abundant protein tightly associated with mtDNA, we can generate singlet oxygen in close proximity to mtDNA and generate the 8-oxoG lesion at high levels in the mitochondrial genome. High levels of 8-oxoG in mtDNA have been associated with mtDNA mutation, deletion, apoptosis, mitochondrial fragmentation, and mitochondrial dysfunction [60,61], and by specifically targeting the mtDNA with this oxidative damage, we can reduce the variables associated with previous methods for damaging mitochondria to determine if persistent oxidative mtDNA damage leads to human pathologies. The ability to damage mtDNA in living cells and organisms with high spatial and temporal control would be ideal for understanding the effects of mtDNA damage on cell fate, tissue injury, and pathophysiology associated with disease.

## Data Availability

Not applicable.
